# 2016 update on APBioNet’s annual international conference on bioinformatics (InCoB)

**DOI:** 10.1186/s12864-016-3362-2

**Published:** 2016-12-22

**Authors:** Christian Schönbach, Chandra Verma, Lawrence Jin Kiat Wee, Peter John Bond, Shoba Ranganathan

**Affiliations:** 1 International Research Center for Medical Sciences, Graduate School of Medical Sciences, Kumamoto University, Kumamoto, 860-0811 Japan; 2 Bioinformatics Institute, Agency for Science, Technology and Research (A∗STAR), Singapore, 138671 Singapore; 3 Institute for Infocomm Research, Agency for Science, Technology and Research (A*STAR), Singapore, 138632 Singapore; 4 Department of Chemistry and Biomolecular Sciences, Macquarie University, Sydney, NSW 2109 Australia

**Keywords:** InCoB, International conference on bioinformatics, APBioNet, Asia-Pacific bioinformatics network

## Abstract

InCoB became since its inception in 2002 one of the largest annual bioinformatics conferences in the Asia-Pacific region with attendance ranging between 150 and 250 delegates depending on the venue location. InCoB 2016 in Singapore was attended by almost 220 delegates. This year, sessions on structural bioinformatics, sequence and sequencing, and next-generation sequencing fielded the highest number of oral presentation. Forty-four out 96 oral presentations were associated with an accepted manuscript in supplemental issues of BMC Bioinformatics, BMC Genomics, BMC Medical Genomics or BMC Systems Biology. Articles with a genomics focus are reviewed in this editorial. Next year’s InCoB will be held in Shenzen, China from September 20 to 22, 2017.

**Electronic supplementary material:**

The online version of this article (doi:10.1186/s12864-016-3362-2) contains supplementary material, which is available to authorized users.

## Introduction

The International Conference on Bioinformatics (InCoB) was held for the first time at Biopolis, Singapore in 2009 and returned in September 21 to 23, 2016 [1] to attract approximately 220 delegates. Singaporean bioinformatics pioneers and APBioNet founders Subramanian Subbiah and Tin Wee Tan highlighted in a special InCoB session 20 years of history and achievements of bioinformatics in Singapore and particularly at the Bioinformatics Centre at NUS. Four parallel tracks accommodated 19 sessions including 96 oral presentations and two sessions with 79 poster presentations.

Five keynotes addressed the latest state of research and advances in genomics transcriptomics and proteomics. Barak Cohen talked about the analysis of combinatorial cis-regulation. Mihaela Zavolan’s talk focused on predicting small RNA targets to learn about gene expression regulatory networks. Vanessa Hayes presented how next generation mapping provides new insights into complex genomic variation of significance to human health and cancer. Sir Tom Blundell’s provided insight into structural bioinformatics and genomic variation: understanding and combating genetic disease and drug resistance and Shoba Ranganathan updated the audience on accelerating the search for the human proteome’s “missing proteins”.

Three medical genomic-themed satellite workshops were conducted at the end of the first conference day: (1) An introduction to transcriptomics and cancer genomics: tools, databases and workflows, (2) Garuda Platform: re-imagining connectivity in biology and medicine and (3) Decoding causative mutations: finding SNPs in a sea of sequenced data.

At the Annual General Meeting on September 21^st^ Shoba Ranganathan delivered the President’s Report on the state of APBioNet and its activities in the past year. The AGM was concluded with the announcement of election results for the Executive Committee (ExCo), term 2016–2018. The list of elected ExCo members and office bearers is available at APBioNet and ISCB affiliates websites [2, 3].

## Manuscript submission and review

Authors had a choice of five journals for submission of original research or software and database articles: BMC Genomics, BMC Bioinformatics, BMC Systems Biology, BMC Medical Genomics, PeerJ, or Journal of Bioinformatics and Computational Biology (JBCB). Of 101 submitted manuscripts 49 (48.5%) were accepted after peer review by at least two members of the Program Committee or external sub-reviewers (Additional file 1) in revised form. Forty-four accepted manuscripts are published in the InCoB2016 supplements of BMC Medical Genomics (6), BMC Systems Biology (5), BMC Genomics (14), BMC Bioinformatics (19) issues, two in PeerJ and three in JBCB. Four manuscripts with top reviewers’ scores were selected for Best Paper Awards (Additional file 2). An overview of the 24 research papers published in the supplement issues of BMC Bioinformatics and BMC Systems Biology is available as introduction in the InCoB2016 supplement of BMC Bioinformatics [4]. The articles included in the BMC Genomics and BMC Medical Genomics supplements are briefly reviewed here.

## NGS informatics

Biomarker discovery is a significant area of biomedical research, where immense transcriptome datasets are analysed and compared. To facilitate this intensive process, Moon and Nakai [5] propose an efficient and accurate ensemble *L*_*1*_-norm support vector machine approach for multi-dimensional data, tested on renal cancer patient data. On the other hand, Hu *et al.* [6] have used support vector machine-based recursive feature elimination approach to zoom in on key single cell brain transcripts for studying neurological diseases.

To rapidly estimate the variation in expression levels of novel transcripts across multiple human and mouse tissues and cell types, Hou *et al.* [7] have developed LocExpress as a freely available webserver, while Ponyared *et al.* [8] have developed the EASP Plus server for mining simple sequence repeat (SSR) markers for plant genetic trait characterization. For targeted gene editing, zinc finger proteins can be engineered to bind to specific DNA sequences. Dutta *et al.* [9] have analysed zinc finger-DNA complex structures and developed a method to predict zinc finger recognition sites for a given DNA nonamer, also implemented as a webserver, Zifpredict_ihbe. Furthermore, Dutta *et al.* [10] have used an ensemble micro neural network approach to decipher DNA-zinc finger interactions and developed a webserver, ZifNN, for designing zinc finger proteins that will preferentially bind to a given DNA sequence.

## Proteomics and proteogenomics

Complementary to genome and transcriptome analysis, proteomics and proteogenomics are increasingly becoming complementary analytical approaches. For comparative proteomic analysis, Goh [11] have developed Fuzzy-FishNET, for robust selection of relevant protein complexes, across patient samples. For identifying novel proteins, especially in disease samples, proteogenomic peptides are mapped directly to genomes. Li *et al.* [12] have thoroughly investigated two popular software tools, X!Tamdem and Comet, for this approach, with several variations and recommend at least two methods for search result validation with separate filtering of known and novel peptides, for improved proteogenomic search sensitivity.

## Genome, epigenome and Gut microbiome analyses

In the era of genome-wide transcriptome studies from different tissues and cell-types, Yarmishyn *et al.* [13] have focussed on the subcellular compartment, the peroxisome, to study mRNA enrichment in a pioneering study, and report a critical cholesterol biosynthesis pathway enzyme as the most enriched transcript. Transcript concentrations vary enormously in a cell, especially as a result of external metabolic stimuli. Kumagai *et al.* [14] report a strong association between RNA degradation patterns and expression dynamics in dendritic cells. Additionally, their comprehensive analysis of patterns of RNA degradation analysis has resulted in a methodology to predict RNA motifs. For scientists working with the model organism, *Arabidopsis thaliana*, Su *et al.* [16] have a develop TEA as an integrated methylome platform, for analysing, annotating and visualizing epigenomic data from large-scale bisulfite-sequencing approaches.

Adaptations to climatic changes and environmental pollutants have a quantifiable effect of the genomes of organisms facing these challenges. Suryavanshi *et al.* [15] have studied the extremophile, *Arabidopsis halleri* growing in heavy-metal contaminated soils and identified a link between evolutionary adaptation and gene copy numbers, permitting the accumulation of toxic metals with a lowering of immune defense response. Weng et al. [17] have studied the evolutionary adaptation of hibernation in non-hibernating frogs by tracking their gut microbiome and concluded that artificial hibernation could result in exposure to pathogenic bacteria, reducing the fitness of this species.

## Disease informatics

Saini *et al.* [18] propose a new genetic algorithm-based gene masking approach to accurately classify cancers, towards discrimination between subtypes and for early detection. A new gene sub-network-based feature selection approach by Doungpan *et al*. was applied to four lung cancer data sets [19]. In this case study the authors’ proposed algorithm improved the classification of sub-networks and agreement between gene and gene-set levels compared to greedy search algorithm.

Lee *et al*. presented a network approach [20] applied to a data set on schizophrenia DNA methylation in the human frontal cortex [21]. The differential methylation analysis revealed both hyper and hypomethylated promoters that may have complementary roles in the development of schizophrenia. Subramanian *et al.* [22] analysed palindromic DNA sequences in breast cancer genomes and noted that a significant number occurring near oncogenes are differentiated, suggesting tumour progression and/or poor prognosis.

Resistance to chemotherapeutic agents has been a serious issue in treating cancer patients. Koh *et al.* [23] have characterized the role of the DNA polymerase alpha subunit B (POLA2) gene in human lung cancer cells. Their results show that the POLA2 knock down is indeed involved in gemcitabine resistance, and suggest that POLA2 may be used as a prognostic biomarker for lung cancer patient outcome. Srinivasulu *et al*. estimated the survival time of 247 patients with glioblastoma multiforme (GBM) in context of miRNA expression profiles [24]. They identified 24 miRNA signatures that are associated with survival time which might have utility in improving GBM therapy. Chen *et al*. applied association rule mining to the immunoinformatics problem of characterizing human influenza virus antigenic evolution [25]. Rules of co-occuring mutations revealed interactions of multiple site mutations in influenza A/H3N2, A/H1N1 and B viruses that contribute to better understanding of antigenic evolution and potential prediction of mutations in HA1.

## Conclusion

With InCoB maturing and a growing interest of having broadly themed bioinformatics conferences for practitioners and developers, next year’s conference will be held in Shenzhen from Sept. 20–22, 2017 [26]. Easychair is expected to open for paper submissions in January 2017.

## Additional files

Additional file 1: List of InCoB2016 Reviewers (PDF 109 kb)
Additional file 2: InCoB2016 Best Paper Awards (PDF 86 kb)
