# Supplementary material for: 2016 update on APBioNet’s annual international conference on bioinformatics (InCoB)
Source: BMC Genomics. 2016 Dec 22;17(Suppl 13):1036. doi: 10.1186/s12864-016-3362-2 (PMC5259860; doi:10.1186/s12864-016-3362-2)
Supplement: Additional file 1: — List of InCoB2016 Reviewers (PDF 109 kb) [file 12864_2016_3362_MOESM1_ESM.pdf]

Additional File 1. List of InCoB2016 Reviewers

We are grateful to the members of InCoB2016 Scientific Program Committee and the sub-reviewers listed in alphabetical order for peer reviewing manuscripts submitted to the InCoB2016 supplement issues of BMC Genomics, BMC Medical Genomics, BMC Bioinformatics, BMC Systems Biology or Journal of Bioinformatics and Computational Biology.

InCoB2016 Scientific Program Committee:

1. Catherine Abbott, Flinders University, Australia
2. Tatsuya Akutsu, Kyoto University, Japan
3. Masanori Arita, National Institute of Genetics, Japan
4. Nicola Armstrong, Garvan Institute of Medical Research, Australia
5. Vladimir Bajic, King Abdullah University of Science and Technology (KAUST), Saudi Arabia
6. Arsen Batagov, A*STAR, Bioinformatics Institute, Singapore
7. Michael Beer, Johns Hopkins University, USA
8. Peter Bond, A*STAR, Bioinformatics Institute, Singapore
9. Edmond Breen, APAF, Ltd. Australia
10. Filippo Castiglione, National Research Council of Italy, Institute for Computing Application, Italy
11. Kun-Mao Chao, National Taiwan University, Taiwan
12. Jonathan Chan, King Mongkut's University of Technology Thonburi, Thailand
13. Michael Charleston, The University of Sydney, Australia
14. Ming Chen, Zhejiang University, PR China
15. Keng-Hwee Chiam, A*STAR, Bioinformatics Institute, Singapore
16. Alan Christoffels, South African Bioinformatics Institute

17. Andrew Dalby, University of Oxford, UK
18. Hao Fan, A*STAR, Bioinformatics Institute, Singapore
19. Mohd Firdaus-Raih, Universiti Kebangsaan Malaysia, Malaysia
20. Stephen Fox, A*STAR, Bioinformatics Institute, Singapore
21. Andrew French, University of Nottingham, UK
22. Bruno Gaeta, University of New South Wales, Australia
23. Ge Gao, Peking University, PR China
24. Pascale Gaudet, Swiss Institute of Bioinformatics, Switzerland
25. Michael Gromiha, Indian Institute of Technology Madras, India
26. Yongqun He, University of Michigan, USA
27. Paul Horton, AIST Computational Biology Research Center, Japan
28. Chia-Lang Hsu, National Taiwan University, Taiwan
29. Fang Hsu, Feng Chia University, Taiwan
30. Wenlian Hsu, Academica Sinica, Taiwan
31. Guang Hu, Soochow University, PR China
32. Yongli Hu, A*STAR, Institute for Infocomm Research, Singapore
33. Hsuan-Cheng Huang, National Yang-Ming University, Taiwan
34. Ming-Jing Hwang, Academia Sinica, Taiwan
35. Ulykbek Kairov, Nazarbayev University, Kazakhstan
36. Asif M. Khan, Perdana University, Malaysia
37. Tsung Fei Khang, University of Malaya, Malaysia
38. Akira Kinjo, Osaka University, Japan
39. Kengo Kinoshita, Tohoku University, Japan
40. Akihiko Konagaya, Tokyo Institute of Technology, Japan
41. Shinji Kondo, National Institute of Polar Research, Japan

42. Anton Kratz, RIKEN Center for Life Science Technologies, Japan
43. Gaurav Kumar, Virginia Commonwealth University, USA
44. Igor V. Kurochkin, A*STAR, Bioinformatics Institute, Singapore
45. Chee Keong Kwoh, Nanyang Technological University, Singapore
46. Jinyan Li, University of Technology Sydney, Australia
47. Jianguo Li, A*STAR, Bioinformatics Institute, Singapore
48. Xiaoli Li, A*STAR, Institute for Infocomm Research, Singapore
49. Lit Hsin Loo, A*STAR, Bioinformatics Institute, Singapore
50. Lloyd Low, Perdana University, Malaysia
51. Suryani Lukman, Khalifa University, UAE
52. Adeel Malik, Perdana University, Malaysia
53. Hiroshi Mamitsuka, Kyoto University, Japan
54. Jan Marzinek, A*STAR, Bioinformatics Institute, Singapore
55. Hideo Matsuda, Osaka University, Japan
56. Sebastian Maurer-Stroh, A*STAR, Bioinformatics Institute, Singapore
57. Bui Quang Minh, Max F. Perutz Laboratories, Austria
58. Lenny Moise, University of Rhode Island, USA
59. Santo Motta, University of Catania, Italy
60. Kenta Nakai, The University of Tokyo, Japan
61. Yasushi Okazakhi, Saitama Medical University, Japan
62. Francesco Pappalardo, University of Catania, Italy
63. Ashwini Patil, The University of Tokyo, Japan
64. Nikolai Petrovsky, Flinders University, Australia
65. Shoba Ranganathan, Macquarie University, Australia
66. Yasubumi, Sakakibara, Keio University, Japan

67. Daniele Santoni, NRC Italy, Institute for System Analysis and Computer Science "Antonio Ruberti", Italy
68. Christian Schönbach, Nazarbayev University, Kazakhstan
69. Tetsuo Shibuya, The University of Tokyo, Japan
70. Adelene Sim, A*STAR, Bioinformatics Institute, Singapore
71. Narayanaswamy Srinivasan, Indian Institute of Science, India
72. Chinh Tran-To Su, Nanyang Technological University, Singapore
73. Durai Sundar, Indian Institute of Technology Delhi, India
74. Yoshihiro Taguchi, Chuo University, Japan
75. Yoichi Takenaka, Osaka University, Japan
76. Tin Wee Tan, National University of Singapore, Singapore
77. Paolo Tieri, National Research Council of Italy, Institute for Applied Mathematics "Mauro Picone", Italy
78. Sissades Tongsima, National Center for Genetic Engineering and Biotechnology, Thailand
79. Chandra Verma, A*STAR, Bioinformatics Institute, Singapore
80. Mauno Vihinen, Lund University, Sweden
81. Lawrence Wee, A*STAR, Institute for Infocomm Research, Singapore
82. Dongqing Wei, Shanghai Jiaotong University, PR China
83. Limsoon Wong, National University of Singapore, Singapore
84. Yingqiu Xie, Nazarbayev University, Kazakhstan
85. Siu-Ming Yiu, The University of Hong Kong, Hong Kong
86. Guang Lan Zhang, Boston University, USA
87. Dongxiao Zhu, Wayne State University, Japan
88. Shanfeng Zhu, Fudan University, PR China

Sub-reviewers (14): Carlo Bianca, Daniel Holdbrook, Roland Huber, Fransiskus X Ivan, Nanying Liang, Le Ou-Yang, Sung-Joon Park, Vinca Prana, Vivek Tanavde, Theerawit Wilaiprasitporn, Min Wu, Peng Yang, Zhuo Zhang and Andrei Zinovyev.
