# Supplementary material for: 2016 update on APBioNet’s annual international conference on bioinformatics (InCoB)
Source: BMC Genomics. 2016 Dec 22;17(Suppl 13):1036. doi: 10.1186/s12864-016-3362-2 (PMC5259860; doi:10.1186/s12864-016-3362-2)
Supplement: Additional file 2: — InCoB2016 Best Paper Awards (PDF 86 kb) [file 12864_2016_3362_MOESM2_ESM.pdf]

Additional File 2. InCoB2016 Best Paper Awards

**Category**	**Best Paper Award**
BMC Genomics	Hou M, Tian F, Jiang S, Kong L, Yang D, Gao G. **LocExpress: a web server for efficiently estimating expression of novel transcripts**. *BMC Genomics*. 2016; **17 Suppl 14**:14.
BMC Medical Genomics	Goh WWB. **Fuzzy-FishNET: A highly reproducible protein complex-based approach for feature selection in comparative proteomics**. *BMC Medical Genomics*. 2016; **17 Suppl 6**:1.
BMC Bioinformatics	Ezzat A, Wu M, Li XL, Kwoh CK. **Drug-target interaction prediction via class imbalance-aware ensemble learning**. *BMC Bioinformatics*. 2016; **17 Suppl 19**:17.<br>Joshi K, Goyal S, Grover S, Jamal S, Singh A, Dhar P, et al. **Novel group-based QSAR and combinatorial design of CK-1δ inhibitors as neuroprotective agents**. *BMC Bioinformatics*. 2016; **17 Suppl 19**:19.
BMC Systems Biology	Lee M, Lee HJ. **DMirNet: inferring direct microRNA-mRNA association networks**. *BMC Systems Biol. 2016*; **10 Suppl 5**:5.
